# Development and Treatment of Severe Lordoscoliosis in a Patient With Noonan Syndrome With Multiple Lentigines (NSML): A Case Report

**DOI:** 10.7759/cureus.101469

**Published:** 2026-01-13

**Authors:** Rui Shian Lee, Fiona Craigen, Athanasios I Tsirikos

**Affiliations:** 1 Paediatric Surgery Department, Royal Hospital for Children and Young People, Edinburgh, GBR; 2 Scottish National Spine Deformity Centre, Royal Hospital for Children and Young People, Edinburgh, GBR

**Keywords:** contraindications to mri, contraindications to tcmeps, intraoperative neuromonitoring (ionm), leopard syndrome, noonan syndrome with multiple lentigines, posterior spinal fusion, scoliosis surgery, syndromic lordoscoliosis

## Abstract

Noonan syndrome with multiple lentigines (NSML) is an allelic variant of Noonan syndrome (NS), which can exhibit multisystemic features, including sensorineural hearing loss (SNHL) and skeletal anomalies. We report on a patient with this rare condition who developed an uncommon syndromic feature of severe lordoscoliosis requiring surgical intervention, whose comorbidities complicated the perioperative course, including the preclusion of magnetic resonance imaging (MRI) and relative contraindication to transcranial motor evoked potentials (TcMEPs). We discuss the use of preoperative computed tomography (CT) and somatosensory evoked potentials (SSEPs) in facilitating the consent process for surgery where MRI and TcMEPs are impracticable, as well as the intraoperative measures taken to minimise complications in this case. We also highlight the importance of multispecialty, multidisciplinary involvement in the management of such patients. In this patient whose severe lordoscoliosis led to persistent back pain and airway compression, posterior spinal fusion achieved satisfactory deformity correction and excellent clinical outcome, which was maintained at follow-up.

## Introduction

Noonan syndrome with multiple lentigines (NSML) is a rare syndrome with only about 200 cases reported in the current literature. It is a part of the RASopathies, a group of genetic conditions affecting the Ras/mitogen-activated protein kinase (Ras/MAPK) signalling pathway, which plays a critical role in cell cycle regulation [1,2]. The effects of abnormalities in this pathway are multisystemic with a widely varied phenotype [1,2]. Specific to NSML, the commonest features include lentigines, dysmorphic facies, hypertrophic cardiomyopathy (HCM), electrocardiogram (ECG) abnormalities and short stature. Less common features include sensorineural hearing loss (SNHL), musculoskeletal anomalies and autoimmune conditions [1,3]. Unlike Noonan syndrome (NS), NSML is not characteristically associated with bleeding diathesis, although there are some reports of haematological abnormalities in these patients [4].

Notably, scoliosis is an uncommon finding amongst musculoskeletal anomalies seen in NSML [1]. We report a unique case of a patient with NSML who underwent posterior spinal fusion for severe syndromic lordoscoliosis causing airway compression. We detail the meticulous, multidisciplinary approach to the perioperative journey in the context of NSML and its comorbidities, in particular cochlear implants for syndromic SNHL, which preclude magnetic resonance imaging (MRI) and multimodal intraoperative neuromonitoring (IONM), specifically transcranial motor evoked potentials (TcMEPs). We also highlight the additional considerations made to aid the consent process for surgery where these standard risk-mitigating tests were impracticable.

To our knowledge, this is the first patient with NSML reported in the current medical literature to undergo scoliosis surgery.

## Case presentation

A female patient aged 15 years and 5 months with NSML was referred to our service with progressive thoracic and lumbar lordoscoliosis. As part of her underlying diagnosis, she had lentigines, short stature (35 kg at 144 cm), delayed puberty, autoimmune hypothyroidism managed with levothyroxine and SNHL requiring bilateral cochlear implants. She was born preterm at 28 weeks and required invasive ventilation and several courses of steroids in the postnatal period.

At presentation, the patient reported persistent back pain, which impacted her daily life and limited her activity levels. A physical examination of her spine showed a severe right thoracic and left lumbar scoliosis with the elevation of the right shoulder, as well as the prominence of the rib cage and paraspinal muscles adjacent to the convexity of the two curves. The waistline was asymmetrical with the prominence of the right side of her pelvis. She had a positive global sagittal balance of her spine with severe thoracic lordosis and compensatory cervical kyphosis. There was no leg length discrepancy and no neurological abnormalities affecting her upper or lower limbs.

**Figure 1:**
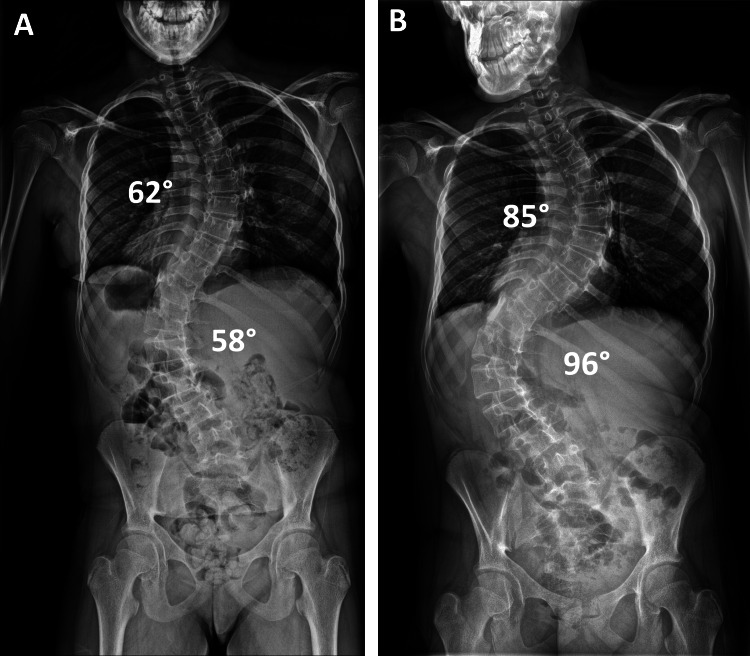
Radiographs showing the rapid progression of scoliosis. Posteroanterior radiographs of the spine taken at initial diagnosis in the patient's local service (A) and when she attended our clinic for the first time (B) show a very significant progression of her thoracic and lumbar scoliosis, which occurred over a period of rapid spinal growth within only eight months.

**Figure 2:**
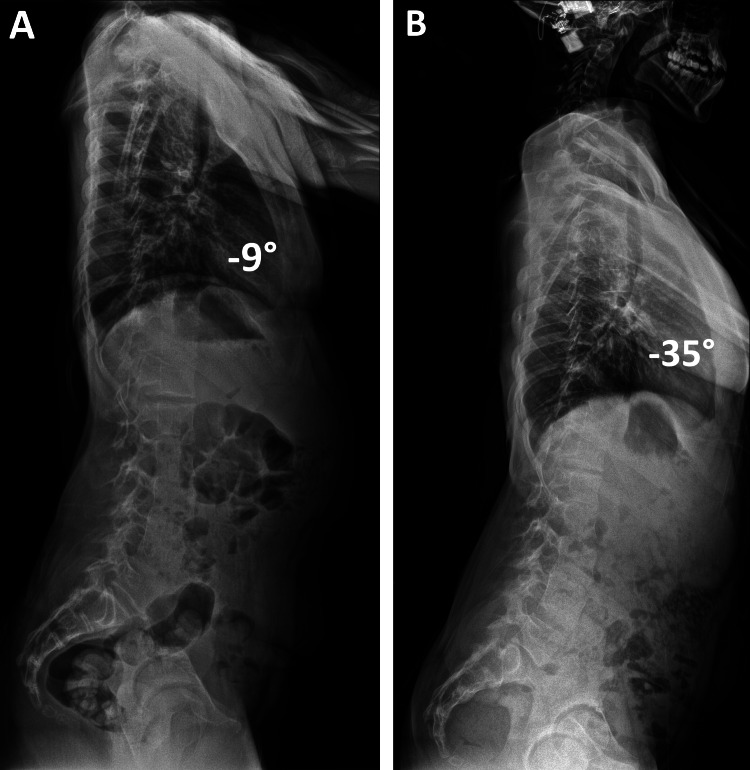
Radiographs showing the rapid progression of thoracic lordosis. Lateral radiographs of the spine taken at initial diagnosis in the patient's local service (A) and when she attended our clinic for the first time (B) show thoracic lordosis that deteriorated rapidly in a period of accelerated pubertal growth spurt, causing spinal penetration into the chest with consequent airway compression. The cochlear implant is noted in situ (B).

X-rays of her spine taken in her local service at age 14 years and 11 months showed a thoracic and lumbar scoliosis, which progressed rapidly within the eight months before her attendance at our clinic (Figure 1). The Cobb angles for thoracic and lumbar scoliosis increased by 23° (62°-85°) and 38° (58°-96°), respectively. In the initial lateral scoliosis X-ray, there was associated mild thoracic lordosis, which also deteriorated from -9° to -35°, and she developed compensatory cervical kyphosis as an attempt to maintain the global sagittal balance of her spine (Figure 2).

**Figure 3:**
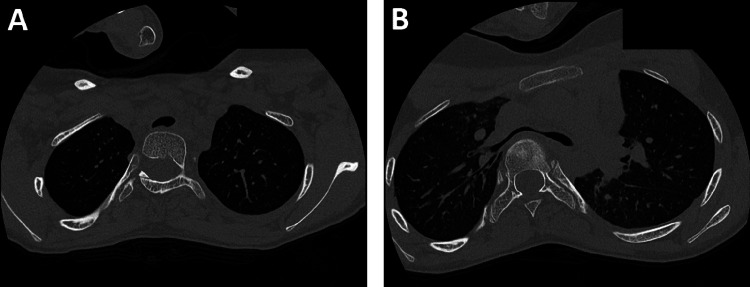
CT showing airway compression from thoracic lordosis. The CT of the chest taken as part of the preoperative assessment shows the compression of the distal trachea (A), carina and left and right main bronchi (B) caused by the vertebral bodies, as, in the presence of marked thoracic lordosis, the anteroposterior diameter of the chest had significantly decreased. CT: computed tomography

The patient underwent a detailed preoperative multidisciplinary assessment, which included respiratory, anaesthetic, cardiac and neurology reviews, as well as blood tests (Table 1). ECG and echocardiography showed no abnormalities, which is of relevance due to the prevalence of cardiovascular anomalies in patients with NSML. Lung function tests showed forced expiratory volume in one second (FEV_1_) of 1.61 L (60% predicted) and forced vital capacity (FVC) of 2.24 L (76% predicted), indicating a restrictive defect with an obstructive component. Transfer factor for carbon monoxide (TLCO) was 61% predicted, showing mild impairment in functional gas exchange, while a computed tomography (CT) scan of her chest showed airway narrowing due to spinal penetration into the chest in the presence of thoracic lordosis (Figure 3). Despite these findings, the patient maintained good respiratory health with no history of recurrent chest infections. The recommendation was for the return of the patient to the intensive care unit (ICU), extubated after surgery, with a low threshold for noninvasive ventilation (NIV) if this was required in the postoperative period.

**Table 1:** Summary of relevant investigations. Laboratory investigations indicated borderline low factor VII, mild derangement in coagulation profile and normal postoperative haemoglobin (Hb). Spirometry and body plethysmography showed a restrictive defect with an obstructive component. PT, prothrombin time; APTT, activated partial thromboplastin time; FEV_1_, forced expiratory volume in one second; FVC, forced vital capacity; TLC, total lung capacity; RV, residual volume; TLCO, transfer factor for carbon monoxide

	Investigations	Results
Haematology	Factor VII:C (0.5-1.5 IU/mL)	0.49 IU/mL
	PT (9.0-12.0 seconds)	13.0 seconds
	APTT (21.0-28.0 seconds)	30.0 seconds
	APTT ratio	1.2
	Preoperative Hb (120-160 g/L)	151 g/L
	Postoperative Hb (120-160 g/L)	121 g/L
Respiratory	FEV_1_	1.61 L (60% predicted)
	FVC	2.24 L (76% predicted)
	FEV_1_/FVC ratio	0.72 (80% predicted)
	TLC	3.63 L (97% predicted)
	RV	1.39 L (169% predicted)
	TLCO	61% predicted

Routine blood tests showed borderline prolonged prothrombin time (PT) at 13.0 seconds and activated partial thromboplastin time (APTT) at 30.0 seconds. A full panel of clotting factors was requested due to the prevalence of abnormal clotting in NS, though this is not established in NSML. The panel showed a borderline low factor VII at 0.49 IU/mL. There was no previous bleeding history. Following advice by our haematologists, IV vitamin K was planned to be given at induction with a low threshold for delivering fresh frozen plasma (FFP) if there were bleeding concerns during surgery.

**Figure 4:**
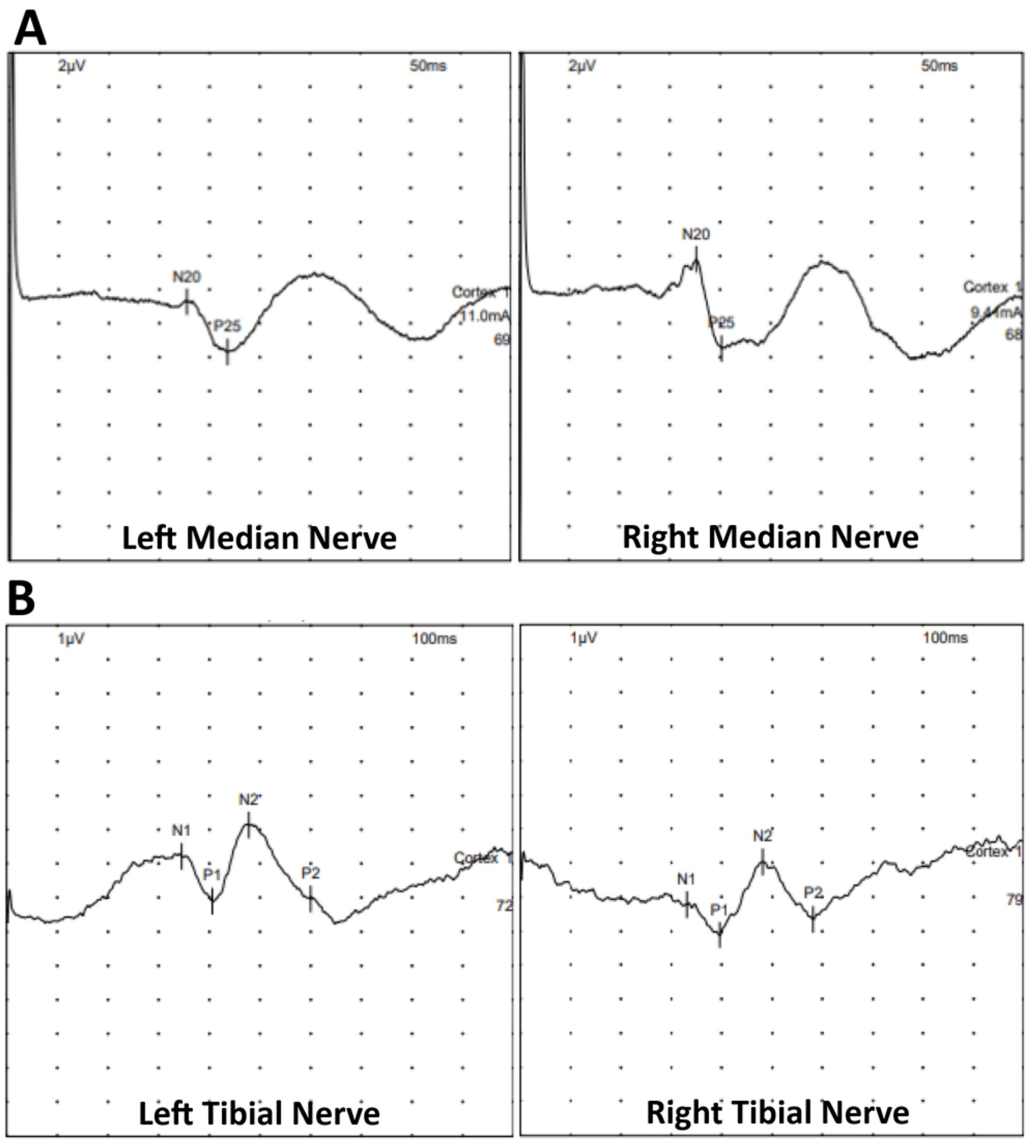
Preoperative baseline SSEPs. Preoperative baseline SSEPs were obtained of the upper (A) and lower limbs (B), and these showed reproducible readings. SSEPs: somatosensory evoked potentials

As was discussed with our MRI team, the presence of cochlear implants precluded a whole spine MRI, which is our standard practice in the presence of a syndromic condition and severe scoliosis in order to detect spinal cord anomalies, hence impacting the risk assessment for intraoperative spinal cord injury. Instead, baseline preoperative somatosensory evoked potentials (SSEPs) were used to support neurological assessment and inform consent for spinal surgery. The SSEPs showed consistent reproducible signals from all four limbs and were considered suitable for IONM (Figure 4). The plan was to record SSEPs and electromyography (EMG) during surgery, as the cochlear implants also constituted a relative contraindication for using motor evoked potentials (MEPs).

**Figure 5:**
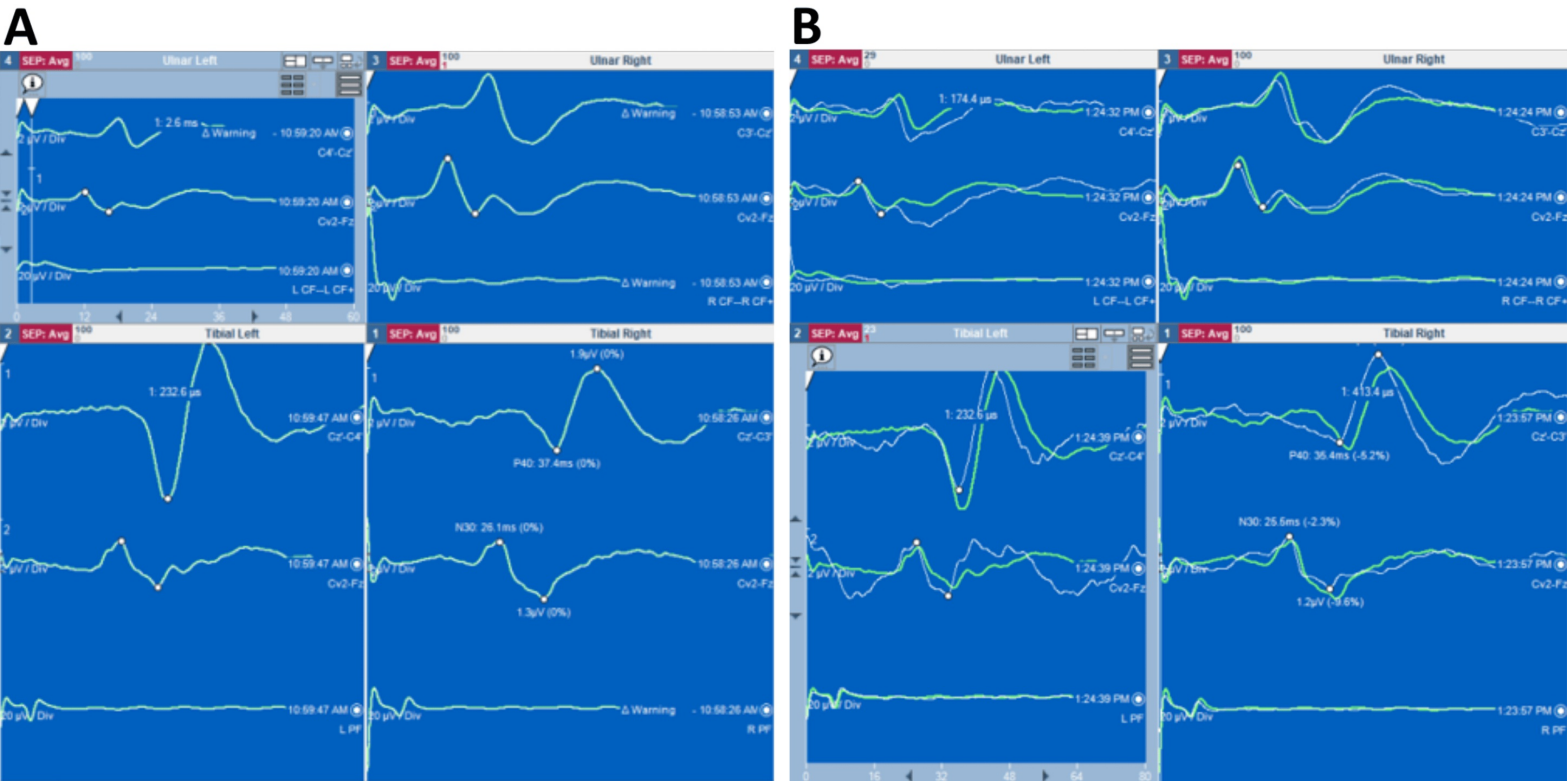
Intraoperative SSEPs. Intraoperative SSEPs were obtained at baseline before skin incision (A), throughout the surgery and at the end of skin closure (B). The green traces show the baseline SSEPs, while the white traces (B) show the final SSEPs at the end of the surgery. No significant changes were recorded. SSEPs: somatosensory evoked potentials

At the age of 15 years and 7 months, the patient underwent a posterior spinal fusion extending from T3 to L4 with the use of pedicle hook, screw and rod instrumentation and a combination of locally harvested autologous and allograft bone. Extensive facetectomies and posterior closing wedge osteotomies were performed at every level across the thoracic and lumbar scoliosis to mobilise the spine and allow for deformity correction. The scoliosis was corrected using rod derotation and a cantilever manoeuvre distally, with focus on restoring thoracic kyphosis. A routine intertransverse interfacetal fusion was performed bilaterally at every level from T3 to L4, followed by the decortication of the posterior elements and an onlay of bone grafts. IONM recorded upper and lower limb cortical and cervical SSEPs and EMGs. Electrodes for TcMEPs were placed to allow the assessment of the long motor tracts only if there were significant changes to the SSEPs. They were not used as SSEPs yielded stable recordings when tested from baseline across to each step of instrumentation placement and following correction manoeuvres throughout the surgery (Figure 5). SSEPs were unchanged in waveform, amplitude and peak latencies between those recorded at the beginning of the surgery and at skin closure; this was reassuring for no significant neurological injury.

Despite the use of IV vitamin K and tranexamic acid, as well as the fact that the surgery took only two hours from skin incision to closure, intraoperative blood volume loss was 35% (800 mL) due to the patient's low body weight of 35 kg. This percentage was obtained by estimating the patient's total blood volume at 65 mL/kg for adolescent women, giving 2275 mL. Cell salvage was used, and this returned 100 mL of autologous blood, which was transfused. No additional blood products were required during or after surgery as the patient remained haemodynamically stable and postoperative haemoglobin was 121 g/L.

In the postoperative period, the patient was transferred to the ICU and made a good recovery post-extubation. She mobilised out of bed on the first postoperative day and received intense respiratory physiotherapy. She returned to the ward the first day after surgery and was discharged home five days later. She was provided with an underarm spinal brace to give additional support during bone graft healing. She continued wearing the brace when mobile out of bed for a period of six months after her surgery.

**Figure 6:**
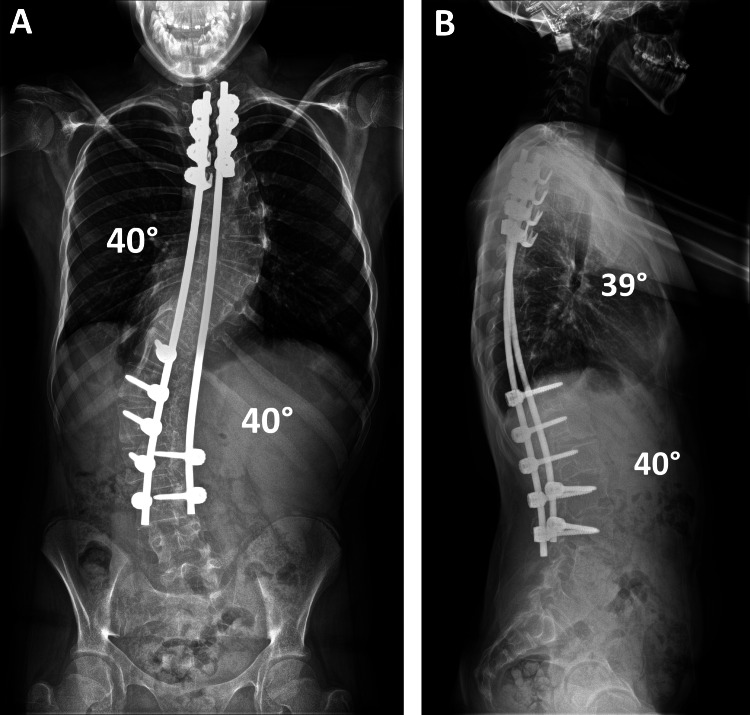
Postoperative radiographs. Posteroanterior radiographs of the spine taken at the last follow-up show a balanced correction of the severe thoracic and lumbar scoliosis, which was maintained over time. Good restoration of the global coronal (A) and sagittal (B) balance of the spine with normal thoracic kyphosis and lumbar lordosis was achieved. At this stage, the patient had returned to her normal level of activities with no complaints of her spine.

Spinal radiographs taken prior to the patient's discharge showed the satisfactory correction of her scoliosis (40° Cobb angle in both thoracic and lumbar spine) and a globally balanced spine in the coronal and sagittal planes with the restoration of thoracic kyphosis to 39°. At the latest follow-up two years after surgery, the patient maintained a balanced spine with no loss of curve correction and no proximal or distal add-on deformity (Figure 6). She had no complaints of back pain and had returned to her normal level of physical activities, including sports. Sequential quality of life assessments using the Scoliosis Research Society (SRS)-22r questionnaire showed incremental improvement in all functional domains from preoperatively to the latest follow-up and a high patient satisfaction (Table 2).

**Table 2:** Scoliosis Research Society (SRS)-22r questionnaire scores over time. The SRS-22r questionnaire is used to measure health-related quality of life (QOL) in patients with scoliosis. It is made up of 22 questions, each scored on a 1-5 Likert scale (higher scores reflect better QOL), covering five domains including function, pain, self-image, mental health and satisfaction. The patient's SRS-22r scores indicate an incremental improvement across all domains over a two-year postoperative follow-up period.

SRS-22r scores	Function	Pain	Self-image	Mental health	Satisfaction	Total
Preoperative	3.6	1.4	1.2	1	-	1.8
6 months post-surgery	3.6	4.6	4.6	4.2	5	4.4
1 year post-surgery	4.4	4.8	4.8	4.4	5	4.68
2 years post-surgery	4.6	4.8	4.9	4.8	5	4.82

## Discussion

NSML is clinically categorised as part of the RASopathies, a group of genetic disorders affecting the Ras/MAPK intracellular signalling pathway, a crucial player in cell cycle regulation, including cell differentiation, growth and senescence [1,2]. Abnormalities in this pathway can produce a wide phenotypical spectrum, including dysmorphic facies, neurocognitive impairment and cardiac, cutaneous, musculoskeletal and ocular anomalies. Other RASopathies include Noonan syndrome, Costello syndrome and cardiofaciocutaneous syndrome [5].

NSML may result from a sporadic mutation or autosomal dominant inheritance. Approximately 90% of cases are due to a mutated *PTPN11* gene on chromosome 12q24.1, encoding for Src homology 2 (SH2) domain-containing protein tyrosine phosphatase-2 (SHP2) [1,3]. Other identified pathogenic gene variants include *RAF1*, *BRAF* and *MAP2K1*. The small proportion of cases without these gene variants is likely associated with as yet undefined genes within the Ras/MAPK pathway [1].

Almost 200 patients with NSML have been reported in the literature, but the actual incidence and population prevalence are unknown [1,3]. It is theorised that the condition is often under-diagnosed or misdiagnosed due to the mild presentation of many of its clinical features and likely also due to its variable phenotype, which often overlaps with other types of RASopathies [3]. 'LEOPARD', from its former name 'LEOPARD syndrome', is a mnemonic acronym for some of its characteristics: lentigines, ECG conduction abnormalities, ocular hypertelorism, pulmonary stenosis, abnormal genitalia, retardation of growth and sensorineural deafness [3]. Other prominent features include HCM, facial dysmorphism, short stature and skeletal anomalies [1,3]. Of these, lentigines are the hallmark feature distinguishing NSML from NS. NSML has a higher incidence rate of SNHL (approximately 20%) than NS (<5%) (wherein conductive hearing loss is more common) and a much greater incidence rate (approximately 80%) of HCM compared to all other RASopathies [5,6].

Chest wall anomalies are the commonest musculoskeletal anomalies in NSML, seen in up to 75% of patients. Scoliosis is a less frequent musculoskeletal finding, alongside conditions such as mandibular prognathism, winging of the scapulae and joint hyperflexibility [3]. In patients with NSML with scoliosis warranting surgery, the potential associated comorbidities should be considered carefully. A multidisciplinary approach is required to formulate a management plan that can minimise surgical complications and optimise recovery.

The MRI of the whole spine is used in patients with severe scoliosis as part of their preoperative assessment to detect neural axis malformations, such as syringomyelia, Chiari malformation, tethered cord or diastematomyelia. Spinal deformity correction in the presence of spinal dysraphism can be associated with increased risk for iatrogenic spinal cord injury [7]. More recent research suggests that on some occasions, intraspinal malformations can be left alone if the preoperative neurological status is intact or stable [8]. There is controversy on the need for preoperative spinal MRI in patients with idiopathic scoliosis, but the presence of neurological deficits, thoracic hyperkyphosis, a syndromic background and an early-onset scoliosis would generally warrant a whole spine MRI [7,9]. There is a lack of data reporting on the spinal MRI findings in patients with NSML. Additionally, the presence of cochlear implants, which the patient had due to SNHL, is either MRI conditional, where not all MRI field strengths are safe, certain parts must be removed prior to MRI and/or the magnet needs securing with a head wrap, or MRI unsafe. Although newer cochlear implant products demonstrate better MRI compatibility, the external components, such as the sound processor and the coil, are always MRI unsafe and must be removed before obtaining an MRI [10,11]. On the basis of these restrictions and with recommendation by the MRI team, this patient did not undergo a spinal MRI as part of her preoperative workup.

IONM is another essential component of scoliosis surgery as it is used to detect new neurological deficits, which can arise intraoperatively. Its use in the context of deformity surgery is preferably multimodal, comprising SSEPs, TcMEPs and EMG, as this is more sensitive and specific in detecting neurological deficits than unimodal IONM [12]. EMG monitors specific spinal nerve roots at risk of injury; SSEPs continuously monitor cortical and cervical responses to peripheral stimulation, albeit signal change can be delayed; TcMEPs detect responses in individual muscle groups to intermittent stimulation at the motor cortex. Of these, TcMEPs are the most sensitive and hence immediately reliable in scoliosis surgery, though not necessarily more specific [13]. Although there is no international protocol on specific intraoperative interventions to be made in response to IONM changes, it is essential to have a clear algorithm that can guide the clinical team's response to a neuromonitoring event during surgery to reduce the risk of permanent neurological damage. At a local hospital level, this necessitates close collaboration between the surgical, anaesthetic and neurophysiology teams [12].

Pertaining to this patient, while there have been case reports on the use of TcMEPs in patients with cochlear implants with no negative outcomes, the current advice from manufacturers and the American Society of Neurophysiological Monitoring is that cochlear implants are a relative contraindication for TcMEPs, citing concerns on neural tissue or device damage [14]. A risk-benefit analysis is recommended to decide on its use during surgery on a case-by-case basis. In this patient who had no neurological deficits and consistent preoperative baseline SSEPs, we decided that electrodes would be sited for recording TcMEPs but only stimulated if there was a significant alteration in the SSEP potentials intraoperatively. All the precautions taken and the meticulous forward planning were explained to the patient and her family and hence facilitated the consent process for surgery.

In addition to the above, we used a hybrid pedicle hook, screw and rod instrumentation technique as opposed to an all-pedicle screw construct for deformity correction. This correction technique reduced surgical time and the neurological risk associated with implant placement as pedicle screws were not used in the upper/mid-thoracic spine, where the risk of spinal cord injury during scoliosis surgery is greatest. The reduced surgical time also minimised the consequent blood loss in a patient who was at high risk of increased blood volume loss due to her low body weight and mild clotting anomalies.

A comprehensive respiratory assessment is included in our standard preoperative spinal pathway, especially in patients who are at high risk of pulmonary compromise, to predict the likelihood of postoperative respiratory complications and formulate a clear treatment plan [15]. This patient had no previous history of respiratory problems, but her scoliosis was associated with severe thoracic lordosis. She underwent lung function tests and a chest CT to investigate potential restrictive and obstructive lung disease [16]. She was found to have extrinsic airway compression due to the penetration of the spinal column into the thorax, which is a risk factor for gas trapping and lobar collapse. As such, the surgery aimed to not only correct the severe scoliosis but also restore normal thoracic kyphosis and global sagittal balance of the spine. NIV was available postoperatively as it is often essential in reducing the risk of chest infection and atelectasis in those with preoperative respiratory complications, including extrinsic bronchial compression, especially in the context of major spinal surgery [16]. Fortunately, it was not required, and the focus of this patient's postoperative care was on early mobilisation out of bed and intensive chest physiotherapy.

Various bleeding disorders have consistently been reported in patients with NS, but there is a lack of data establishing a correlation between NSML and bleeding diathesis [17]. A mouse model study showed that NSML with SHP2 loss of function had a shear-dependent increase in platelet activation, which conversely increases the risk of thrombotic events [4]. Due to the prevalence of bleeding disorders in NS and the limited information available on NSML, advice was sought from our haematology colleagues, who suggested a detailed bleeding history and a full panel of clotting factors on top of routine preoperative blood tests. The patient had marginally prolonged PT and APTT with borderline low factor VII but no abnormal bleeding history. The use of tranexamic acid and IV vitamin K at induction may have helped reduce intraoperative blood loss. We also used cell salvage, which recirculates the patient's own blood and is our routine practice during major spinal deformity surgery. Attention was taken to respect the soft tissues during exposure and expedite the surgery in order to reduce intraoperative blood loss. These measures allowed us to avoid the need for allogeneic blood transfusion during or after surgery despite the fact that the patient lost 35% of her blood volume.

Other considerations that were not encountered in this patient but are important to recognise in association with NSML are cardiac anomalies and autoimmune diseases. HCM is the commonest cardiovascular abnormality in NSML, presenting in 80%-90% of patients often from a very young age and posing as a significant risk factor for adverse cardiac events, including death [5,18]. It has pertinent implications on surgery and anaesthesia, both of which can promote haemodynamic instability and lead to the exacerbation of left ventricular outflow tract obstruction [19]. Thus, patients with NSML should have a thorough preoperative assessment of their cardiovascular function with specialist input to best prevent adverse outcomes. Additionally, there is emerging evidence in the literature indicating an association between RASopathies and autoimmune diseases, including systemic lupus erythematosus, coeliac disease and autoimmune thyroiditis [2]. There is limited information on the effect of autoimmune disease on scoliosis surgery, but this would be expected to increase the risk of infection after a major procedure. Existing literature reports that adult patients with autoimmune conditions who underwent spinal deformity correction had more postoperative complications and worse patient-reported clinical outcomes [20].

## Conclusions

Our case report presents the first patient with NSML reported in the current literature to undergo scoliosis surgery and their clinical outcomes. We highlight the considerations and precautions taken to manage such a case with multiple uncertainties throughout the perioperative course, including the use of CT imaging and SSEPs in obtaining consent for surgery. We also emphasise that multidisciplinary involvement is paramount in these instances to optimise perioperative care, minimise the risk of complications and enhance postoperative recovery. In this patient with NSML and severe, rapidly progressive lordoscoliosis causing pain, airway compression and impaired lung function, spinal surgery achieved successful deformity correction and a satisfactory clinical outcome with high patient satisfaction, which was maintained at follow-up.
